# A Fourth-Order Compact Finite Difference Scheme for Solving the Time Fractional Carbon Nanotubes Model

**DOI:** 10.1155/2022/1426837

**Published:** 2022-03-02

**Authors:** N. H. Sweilam, Khloud R. Khater, Zafer M. Asker, Waleed Abdel Kareem

**Affiliations:** ^1^Faculty of Science, Cairo University, Giza, Egypt; ^2^High Institute for Engineering and Technology, Minya, Egypt; ^3^Faculty of Science, Suez University, Suez, Egypt

## Abstract

In this work, we deal with unsteady magnetohydrodynamic allowed convection inflow of blood with a carbon nanotubes model; the single and multiwalled carbon nanotubes of human blood are used as a based fluid. Two numerical methods used to study this model are the weighted average finite difference method and the nonstandard compact finite difference method. The proportional Caputo hybrid operator has been used to fractionalize the proposed model. Stability analysis has been construed by a kind of John von Neumann stability analysis. Numerical results are presented in diverse graphs, which manifest that the method is successful in solving the proposed model.

## 1. Introduction

Fractional calculus (FC) is a generalization of the integer-order calculus. In fractional calculus, researchers try to solve problems with *α*-order derivatives and integrals, where there are several definitions for derivatives of order *α* [1, 2]. The most common derivatives are the Riemann-Liouville [3], Caputo [3], Caputo–Fabrizio [4], and the proportional Caputo hybrid [5] formulations. In scopes of fluid dynamic and engineering, most nanofluids waft issues are typically nonlinear character, and it is believed that the fractional-order methods are the best suited models to act for such studies comparatively different conventional methods [6].

Oberlin et al. [7] were the first to initiate the carbon nanotubes (CNTs) as nanoparticles in 1976. CNTs are one of the nanomaterials that are vastly used in such parts in the last years. They have got more attention because of their unrivalled advantages [8]. CNTs have extraordinary conductivity which helps them to form a network of conductive tubes. CNTs have also been used for thermal defence as thermal boundary materials. In 1995, a novel magnificence of warmth transferring fluids that may be engineered via placing metal nanoparticles in conventional heat transfer fluids became initiated by Choi [9]. With the expansion of nanotechnology, many nanomaterials were developed and utilized in the industry. Khan et al. [10] discussed the slip waft of Eyring-Powell nanoliquid film containing graphene nanoparticles. 3D nanofluid waft with heat and mass transferring analysis is over a linear straight floor with convective boundary conditions.

Khalid et al. [11] investigated a case of effects of MHD human blood going with the flow in porosity of the waist CNTs and thermal evaluation. The case is stated and solved for an analytical solution by the usage of the Laplace transform method. Khan [12] investigated the Atangana–Baleanu fractional derivative to blood flow in nanofluids possessing without local and without singular kernels, and it was utilized to blood of nanofluid. Meyer et al. [13] discussed the convective warmth transferring fecundity experimentally of watery deferrals for the multiple-walled CNTs (MWCNTs) flowing horizontal straight tube.

Wang et al. [14, 15] have paid vital interest to the CNTs with various consequences together with heat transfer, thermal conductivity, thermal radiation, the porosity of the medium, and so forth. Qureshi et al. [16] discussed a fractional model for the concentration system of blood ethanol with real data application, where they used the Atangana–Baleanu–Caputo and the Caputo–Fabrizio fractional operators. Saqib et al. [17] solved the fractional derivative nonsingular and local kernel to enhance heat transfer in CNT nanofluid over a sloping plate, and the exact solution expressed analytically for velocity and temperature profiles by the Laplace transformation technique.

Kalita et al. [18] studied a few applications for a vertical tube of CNTs and the porosity of the medium (human blood) flow in the appearance of thermal irradiation and chemical response of first order. Inside this tube, single-walled CNT (SWCNT) and MWCNT were replaced with blood as a based fluid.

The flow problem and its time-fractional form are given in Section 2. Preliminaries of the fractional derivatives definitions are given in Section 3. Moreover, the nonstandard finite difference method (NSFDM) and the nonstandard compact finite difference method (NSCFDM) are given in Section 4. We developed the weighted average nonstandard compact finite difference method (WANSCFDM) for the nanofluid CNTs equations in Section 5. Stability analyses of these schemes are given in Section 6. Numerical solutions for the nanofluid CNTs equations are graphically reported in Section 7. Finally, the conclusions are given in Section 8.

## 2. The Flow Problem and Its Fractional Form

Consider the unstable transportation inflow of human blood CNT-based nanofluid through a columnar platelet with isothermal heat *T*_*inf*_ (ambient heat). The nanofluid is supposed to be with electric carrying with a regular magnetic domain *B* with intensity *B*_0_ utilized in the way vertical to the laminar [11]. The half-space laminar is included in the porosity of the medium satiated with human blood as base nanofluids containing both SWCNT and MWCNT. Let the based fluid and CNTs be in thermal balance, and no slip happens between them; at first, at time *t*=0, the nanofluid and the lamina are stable with constant heat *T*_*inf*_. As in [11], after a small interval of time *t* > 0, the plate vibrates with *V*=*U*_0_*H*(*t*)cos(*Qt*), and the ambient field of temperature of the plate *T*_*inf*_ rises to *T*_*w*_. The temperature and velocity fields equations are

(1) $$\begin{array}{c} \frac{\partial u\left(\xi ,t\right)}{\partial t}=\frac{1}{\phi_{1}}\left(1+\frac{1}{\gamma }\right)\frac{\partial^{2}u\left(\xi ,t\right)}{\partial \xi^{2}}-\left(\frac{M}{\phi_{2}}+\frac{\phi_{1}}{K}\right)u\left(\xi ,t\right)+Gr\phi_{3}\theta \left(\xi ,t\right), \end{array}$$

(2) $$\begin{array}{c} \frac{\partial \theta \left(\xi ,t\right)}{\partial t}=\frac{\phi_{5}}{\mathrm{Pr}\phi_{4}}\frac{\partial^{2}\theta \left(\xi ,t\right)}{\partial \xi^{2}},\;t>0,\xi \in \left(0,l\right), \end{array}$$

with the following boundary and initial conditions:

(3) $$\begin{array}{c} u\left(\xi ,0\right)=0,\;\theta \left(\xi ,0\right)=0,\;\forall \xi >0,\\ u\left(0,t\right)=H\left(t\right)\text{cos}\left(Qt\right),\;\theta \left(0,t\right)=1,\text{ for }t\geq 0,\\ u\left(l,t\right)=0,\theta \left(l,t\right)=0,\forall t>0, \end{array}$$

where

(4) $$\begin{array}{c} M=\frac{\sigma_{f}v_{f}B_{0}^{2}}{\rho_{f}U_{0}^{2}},\;K=\frac{k_{1}U^{2}}{v_{f}^{2}},\;Pr=\frac{v_{f}{\left(\rho C_{p}\right)}_{f}}{k_{f}}\text{ and }Gr=\frac{gv_{f}\beta_{f}\left(T_{w}-T_{\infty }\right)}{U_{0}^{3}}, \end{array}$$

where *ϕ*_*r*_, (*r*=1,2,…, 5) are the constant terms:

(5) $$\begin{array}{c} \phi_{1}={\left(1-\phi \right)}^{2.5}\left[\left(1-\phi \right)+\phi \frac{\rho_{c}}{\rho_{f}}\right],\\ \phi_{2}=1+\left(1-\phi \right)\left\{\frac{3\phi \left(\sigma_{c}/\sigma_{f}-1\right)}{\left(\sigma_{c}/\sigma_{f}+2\right)-\phi \left(\sigma_{c}/\sigma_{f}-1\right)}\right\},\\ \phi_{3}=\left\{\frac{\left(1-\phi \right)+\phi {\left(\rho \beta \right)}_{c}/{\left(\rho \beta \right)}_{f}}{\left(1-\phi \right)+\phi {\left(\rho \right)}_{c}/{\left(\rho \right)}_{f}}\right\},\\ \phi_{4}=\left(1-\phi \right)+\phi \frac{{\left(\rho C_{p}\right)}_{c}}{{\left(\rho C_{p}\right)}_{f}},\;\phi_{5}=\frac{k_{nf}}{k_{f}},\\ k_{nf}=k_{f}\left\{\frac{1-\phi +2\phi \left(k_{c}/k_{c}-k_{f}\right)\ln\left(k_{c}+k_{f}/2k_{f}\right)}{1-\phi +2\phi \left(k_{f}/k_{c}-k_{f}\right)\ln\left(k_{c}+k_{f}/2k_{f}\right)}\right\}. \end{array}$$

The time-fractional forms of equations (1) and (2) are given:

(6) DCPCtαuξ,t=A∂2uξ,t∂ξ2−Luξ,t+Cθξ,t,

(7) DCPCtαθξ,t=F∂2θξ,t∂ξ2,t>0,ξ≥0,

where ^*CPC*^*D*_*t*_^*α*^(*ξ*, *t*) is the constant proportional Caputo time-fractional operator [19], for the same boundary and initial condition (3), where A, C, F, and *L* are the constants:

(8) $$\begin{array}{c} A=\frac{1}{\phi_{1}}\left(1+\frac{1}{\gamma }\right),C=Gr\phi_{3},F=\frac{\phi_{5}}{\mathrm{Pr}\phi_{4}}\text{ and }L=\left(\frac{M}{\phi_{2}}+\frac{\phi_{1}}{K}\right). \end{array}$$

The following analytical solutions are a special case for the temperature and velocity fields by taking *Qt*=0 which agree with the impulsive motion of the plate [11],

(9) $$\begin{array}{c} \theta \left(\xi ,t\right)=erfc\left[\frac{\xi }{2}\sqrt{\frac{b_{0}}{t}}\right],\\ u\left(\xi ,t\right)=\frac{1}{2}\left(e^{-\xi \sqrt{a_{1}L}}erfc\left[\frac{\xi }{2}\sqrt{\frac{a_{1}}{t}}-\sqrt{Lt}\right]+e^{\xi \sqrt{a_{1}L}}erfc\left[\frac{\xi }{2}\sqrt{\frac{a_{1}}{t}}+\sqrt{Lt}\right]\right)\\ +b_{1}\left(e^{-\xi \sqrt{a_{1}L}}erfc\left[\frac{\xi }{2}\sqrt{\frac{a_{1}}{t}}-\sqrt{Lt}\right]\left(\frac{t}{2}-\frac{\xi }{4}\sqrt{\frac{a_{1}}{L}}\right)+e^{\xi \sqrt{a_{1}L}}erfc\left[\frac{\xi }{2}\sqrt{\frac{a_{1}}{t}}+\sqrt{Lt}\right]\left(\frac{t}{2}+\frac{\xi }{4}\sqrt{\frac{a_{1}}{L}}\right)\right)\\ -b_{1}\left(\left(t+\frac{b_{0}\xi^{2}}{2}\right)erfc\left[\frac{\xi }{2}\sqrt{\frac{b_{0}}{t}}\right]-\xi \sqrt{b_{0}}\sqrt{\frac{t}{\pi }}e^{-b_{0}\xi^{2}/4t}\right),\\ \text{when}\\ a_{0}=\frac{\gamma }{1+\gamma },\;a_{1}=a_{0}\phi_{1},\;b_{0}=\frac{Pr\phi_{3}}{\phi_{5}},\;b_{1}=\frac{a_{1}Gr\phi_{1}\phi_{2}}{b_{0}-1}. \end{array}$$

The parameters that appeared in the model are given in Table 1.

The function *erfc* is a complementary error function; it is widely used in statistical computations, for instance, where it is also known as the standard normal cumulative probability. The complementary error function is defined as [20]

(10) $$\begin{array}{c} erfcf\left(t\right)=1-erff\left(t\right). \end{array}$$

## 3. Basic Preliminaries

We are going to reminiscence several important definitions for fractional derivatives. Caputo's fractional-order derivative for 0 < *α* < 1 and Γ be the Euler gamma function of a differentiable function *f*(*t*) and is defined as follows [3]:

(11)  D0Ctαft=1Γ1−α∫0t(f'τt−τ−αdτ  .

The Riemann-Liouville integral, where *α* > 0 and *f*(*t*) is an integrable function, is defined by [3] as

(12)  I0RLtαft=1Γα∫0tt−τα−1fτdτ  .

The hybrid fractional operator is a new fractional operator that is defined by combining the proportional definition, Caputo, and Riemann-Liouville definitions (Baleanu et al. [19]):

(13)  D0CPCtαft=1Γ1−α∫0t(K1αfτ+K0αf′τ)t−τ−α∫0tdr=K1αI0RLt1−αfτ+K0αD0Ctαfτ,

where *K*_0_(*α*), *K*_1_(*α*) are the constants ([21]) defined with respect to time and depending only on the parameter *α*. As in [19], consider the kernels as follows: *K*_0_(*α*)=*αS*^(1 − *α*)^, *K*_1_(*α*)=(1 − *α*)*S*^*α*^, where S is the constant; in our model, it refers to the value of time in the numerical solutions.

## 4. Numerical Methods

### 4.1. NSFDM

In this part, we introduce several comments related to the NSFDM, first proposed by Mickens [22]. The derivative term of the forward method *du*/*dt* is substituted by *u*(*t*+*k*) − *u*(*t*)/*k*, where *k*=Δ*t* is the step size and *k*⟶0; in the Mickens schemes, this term is substituted by *u*(*t*+*k*) − *u*(*t*)/*ϕ*(*k*), where *ϕ*(*k*) is a continuous function of step size k, where the function *φ*(k) satisfies the following conditions:

(14) $$\begin{array}{c} \phi \left(k\right)=k+O\left(k^{2}\right),\;0<\phi \left(k\right)<1,\;k⟶0. \end{array}$$

In the centered method, the derivative term *d*^2^*u*/*dξ*^2^ is substituted by *u*(*ξ*+*h*) − 2*u*(*ξ*)+*u*(*ξ* − *h*)/(*h*)^2^, where h=Δξ is the step size; in the schemes of Mickens, this term is substitute by *u*(*ξ*+*h*) − 2*u*(*ξ*)+*u*(*ξ* − *h*)/(*ψ*(*h*))^2^, where *ψ*(*h*)=*h*+*O*(*h*^2^). Let *Z* and N are the two positive integers, the mesh points have the coordinates *ξ*_*i*+1_=*ξ*_*i*_+*h*, (*i*=0,1,…, *N*) and *t*_*j*+1_=*t*_*j*_+*k*, (*j*=0,1,…, *Z*), and the values of the solution *u*(*ξ*, *t*) on these grid points are *u*(*ξ*_*i*_, *t*_*j*_) ≡ *u*_*i*_^*j*^, where *h*=*l*/*N* and *k*=*T*/*Z*. The forward NSFD formula for the first order of the time and the centered NSFD formula for the second order of space will be

(15) $$\begin{array}{c} \frac{\partial u}{\partial t}=\frac{u_{i}^{j+1}-u_{i}^{j}}{\phi \left(k\right)}+O\left(\phi \left(k\right)\right), \end{array}$$

(16) $$\begin{array}{c} \frac{\partial^{2}u}{\partial \xi^{2}}=\left(\frac{u_{i-1}^{j}-2u_{i}^{j}+u_{i+1}^{j}}{{\left(\psi \left(h\right)\right)}^{2}}\right)+O\left(\psi {\left(h\right)}^{2}\right), \end{array}$$

for more details ([14]).

### 4.2. NSCFDM

The expansion of Taylor is considered a very helpful tool for the derivation of higher-order approximation to derivatives of all orders. Our advantage in this work is to use the higher-order nonstandard finite difference formula for the spatial discretization of the problems, so as to create an estimate based on the step size 2*ψ*(*h*) through the Taylor series expansion ([2]),

(17) $$\begin{array}{c} \left(u\xi_{i}+2\psi \left(h\right)\right)=\sum_{n=0}^{\infty }\frac{{\left(2\psi \left(h\right)\right)}^{n}}{n!}u^{n}\left(\xi_{i}\right),\\ u\left(\xi_{i}-2\psi \left(h\right)\right)=\sum_{n=0}^{\infty }{\left(-1\right)}^{n}\frac{{\left(2\psi \left(h\right)\right)}^{n}}{n!}u^{n}\left(\xi_{i}\right). \end{array}$$

A better approximation can be gained by combining these two assessments using the process called Richardson extrapolation. We will deduce that the fourth-order centered nonstandard finite difference scheme for the second derivative will be

(18) $$\begin{array}{c} \frac{\partial^{2}u}{\partial \xi^{2}}=\left(\frac{-\left(1/12\right)u_{i-2}^{j}+\left(4/3\right)u_{i-1}^{j}-\left(5/2\right)u_{i}^{j}+\left(4/3\right)u_{i+1}^{j}-\left(1/12\right)u_{i+2}^{j}}{{\left(\psi \left(h\right)\right)}^{2}}\right)+O\left(\psi {\left(h\right)}^{4}\right). \end{array}$$

## 5. WANSCFDM

Now, we will use the WANSCFD scheme to obtain the discretization formulas for the temperature and velocity equations. For getting the discretization formulas of equations (4) and (5), we need to substitute the WANSCFD method of the centered formula for space (20) into equations (6) and (7), where *ω* is the weighting factor:

(19)  DCPCtαuξ,t| i,j=Aω−1/12ui−2j+4/3ui−1j−5/2uij+4/3ui+1j−1/12ui+2jψh2+A1−ω−1/12ui−2j+1+4/3ui−1j+1−5/2uij+1+4/3ui+1j+1−1/12ui+2j+1ψh2−Luij+Cθij.

(20)  DCPCtαθξ,t| i,j=Fω−1/12θi−2j+4/3θi−1j−5/2θij+4/3θi+1j−1/12θi+2jψh2+F1−ω−1/12θi−2j+1+4/3θi−1j+1−5/2θij+1+4/3θi+1j+1−1/12θi+2j+1ψh2.

Equations (13) and (14) are the WANSCFD schemes for the temperature and velocity fields. At the case of *ω*=1, we have the forward Euler fractional quadrature scheme, and if we put *ω*=1/2, we get Crank-Nicholson fractional quadrature scheme, but at *ω*=0, the scheme is called totally implicit, which have been studied, e.g., in [23].

Our aim in the current study is to introduce numerical solutions of time-order fractional for equations (6) and (7) with the new derivative operator (hybrid operator) (Baleanu et al. [24]), which is discretized as follows:

(21) DCPCiαuξ,t|i,j=K1α0RLIt1−αuξ,t|i,j+K0α0CDtαuξ,t|i,j.

(22) DCPCiαθξ,t|i,j=K1α0RLIt1−αθξ,t|i,j+K0α0CDtαθξ,t|i,j.

Here, CPC stands for constant proportional Caputo derivative [5]. The discretization of time-order fractional for the Riemann-Liouville operator is given by

(23) D0RLt1−α uξ,t| i,j=δt1−αuij+Oφkp,

where

(24) $$\begin{array}{c} \delta_{t}^{1-\alpha }u_{i}^{j}\equiv \frac{1}{{\left(\varphi \left(k\right)\right)}^{1-\alpha }}\sum_{k=0}^{\left[t_{j}/k\right]}\left(W_{k}^{\left(1-\alpha \right)}u\left(\xi_{i},t_{j}-k\right)\right)=\frac{1}{{\left(\varphi \left(k\right)\right)}^{1-\alpha }}\sum_{k=0}^{m}W_{k}^{\left(1-\alpha \right)}u_{i}^{j-k}. \end{array}$$

The fraction [*t*_*j*_/*k*] means the integer part of *t*_*j*_/*k* and the parameter *p* represents the order of approximation which are dependent on the choice of *W*_*k*_^(1 − *α*)^. The above expression is not the only one because there are different expressions of the weights *W*_*k*_^(*α*)^ [25]. The coefficients *W*_*k*_^(*α*)^ can be evaluated by the recursive formula:

(25) $$\begin{array}{c} W_{k}^{\left(\alpha \right)}=\left(1-\frac{\alpha +1}{k}\right)W_{k-1}^{\left(\alpha \right)},W_{0}^{\left(\alpha \right)}=1, \end{array}$$

where the discretization of time-fractional order of Caputo derivative is given by

(26)  DCtαuξ,t=k1−αφkΓ2−α∑k=0muij+1−k−uij−kk+1 1−α−k1−α,

where 0 < *α* < 1; by substituting equations (25), (26), and (28) into equations (21) and (22), we will get the time-order fractional discretization of hybrid derivative for temperature and velocity equations:

(27) $$\begin{array}{c} K_{1}\left(\alpha \right)\frac{1}{{\left(\varphi \left(k\right)\right)}^{1-\alpha }}\sum_{k=0}^{m}W_{k}^{\left(1-\alpha \right)}u_{i}^{j-k}+K_{0}\left(\alpha \right)\frac{{\left(k\right)}^{1-\alpha }}{\varphi \left(k\right)\Gamma \left(2-\alpha \right)}\sum_{k=0}^{m}\left(u_{i}^{j+1-k}-u_{i}^{j-k}\right)\\ \left(k+1\right){\;}^{\left(1-\alpha \right)}-k^{\left(1-\alpha \right)}-A\omega \left\{\frac{-\left(1/12\right)u_{i-2}^{j}+\left(4/3\right)u_{i-1}^{j}-\left(5/2\right)u_{i}^{j}+\left(4/3\right)u_{i+1}^{j}-\left(1/12\right)u_{i+2}^{j}}{{\left(\psi \left(h\right)\right)}^{2}}\right\}\\ -A\left(1-\omega \right)\left\{\frac{-\left(1/12\right)u_{i-2}^{j+1}+\left(4/3\right)u_{i-1}^{j+1}-\left(5/2\right)u_{i}^{j+1}+\left(4/3\right)u_{i+1}^{j+1}-\left(1/12\right)u_{i+2}^{j+1}}{{\left(\psi \left(h\right)\right)}^{2}}\right\}\;\\ +Lu_{i}^{j}-C\theta_{i}^{j}=T_{i}^{j}, \end{array}$$

(28) $$\begin{array}{c} K_{1}\left(\alpha \right)\frac{1}{{\left(\varphi \left(k\right)\right)}^{1-\alpha }}\sum_{k=0}^{m}W_{k}^{\left(1-\alpha \right)}\theta_{i}^{j-k}+K_{0}\left(\alpha \right)\frac{{\left(k\right)}^{1-\alpha }}{\varphi \left(k\right)\Gamma \left(2-\alpha \right)}\sum_{k=0}^{m}\left(\theta_{i}^{j+1-k}-\theta_{i}^{j-k}\right)\\ \left(k+1{\text{)}}^{\left(1-\alpha \right)}-k^{\left(1-\alpha \right)}\right)-F\omega \left\{\frac{-\left(1/12\right)\theta_{i-2}^{j}+\left(4/3\right)\theta_{i-1}^{j}-\left(5/2\right)\theta_{i}^{j}+\left(4/3\right)\theta_{i+1}^{j}-\left(1/12\right)\theta_{i+2}^{j}}{{\left(\psi \left(h\right)\right)}^{2}}\right\}\\ -F\left(1-\omega \right)\left\{\frac{-\left(1/12\right)\theta_{i-2}^{j+1}+\left(4/3\right)\theta_{i-1}^{j+1}-\left(5/2\right)\theta_{i}^{j+1}+\left(4/3\right)\theta_{i+1}^{j+1}-\left(1/12\right)\theta_{i+2}^{j+1}}{{\left(\psi \left(h\right)\right)}^{2}}\right\}=T_{i}^{j}, \end{array}$$

where *T*_*i*_^*j*^ is the truncating error. More details for discretization in fractional calculus can be found in previous studies such as [24, 25].

## 6. Stability Analysis

To check the stability of schemes (29) and (30), we applying a kind of the Jon von Neumann method [22, 24] by considering systems (4) and (5) can be written in the following form:

(29)  DCPCtαu−Auξξ+Lu−Cθ=0,

(30)  DCPCtαθ−Fθξξ=0,t>0,ξ≥0.

Writing this system in a matrix form is as follows:

(31) Y1DCPCtαX+Y2Xξξ+Y3X=0,X=uθ,Y1=1001,Y2=−A00−F,Y3=L−C00,

and the above system (31) can be formed using the WANSCFD method as follows:

(32) $$\begin{array}{c} Y_{1}\left[K_{1}\right.\left(\alpha \right)\frac{1}{{\left(\varphi \left(k\right)\right)}^{1-\alpha }}\sum_{k=0}^{m}W_{k}^{\left(1-\alpha \right)}X_{i}^{j-k}+K_{0}\left(\alpha \right)\frac{{\left(k\right)}^{1-\alpha }}{\varphi \left(k\right)\Gamma \left(2-\alpha \right)}\sum_{k=0}^{m}\left(X_{i}^{j+1-k}-X_{i}^{j-k}\right)\left(\left({\left(k+1\right)}^{\left(1-\alpha \right)}-k^{\left(1-\alpha \right)}\right)\right]\\ \;+Y_{2}\omega \left\{\frac{-\left(1/12\right)X_{i+2}^{j}+\left(4/3\right)X_{i+1}^{j}-\left(5/2\right)X_{i}^{j}+\left(4/3\right)X_{i-1}^{j}-\left(1/12\right)X_{i-2}^{j}}{{\left(\psi \left(h\right)\right)}^{2}}\right\}\\ +Y_{2}\left(1-\omega \right)\left\{\frac{-\left(1/12\right)X_{i+2}^{j+1}+\left(4/3\right)X_{i+1}^{j+1}-\left(5/2\right)X_{i}^{j+1}+\left(4/3\right)X_{i-1}^{j+1}-\left(1/12\right)X_{i-2}^{j+1}}{{\left(\psi \left(h\right)\right)}^{2}}\right\}+Y_{3}X_{i}^{j}=0. \end{array}$$

Applying the mathematical required steps system (32) will take the following form:

(33) $$\begin{array}{c} A_{1}X_{i+2}^{j+1}+A_{2}X_{i+1}^{j+1}+A_{3}X_{i}^{j+1}+A_{4}X_{i-1}^{j+1}+A_{5}X_{i-2}^{j+1}=Z_{1}X_{i+2}^{j}+Z_{2}X_{i+1}^{j}+Z_{3}X_{i}^{j}+Z_{4}X_{i-1}^{j}+\\ {+Z}_{5}X_{i-2}^{j}+s\sum_{k=1}^{m-1}\left(W_{k}^{\left(1-\alpha \right)}X_{i}^{j-k}+f\sum_{k=1}^{m-1}\left(X_{i}^{j+1-k}-X_{i}^{j-k}\right)\right)z_{k}, \end{array}$$

where

(34) $$\begin{array}{c} A_{1}=A_{5}=\frac{Y_{2}\left(1-\omega \right)}{12{\left(\psi \left(h\right)\right)}^{2}},A_{2}=A_{4}=\frac{-4Y_{2}\left(1-\omega \right)}{3{\left(\psi \left(h\right)\right)}^{2}},A_{3}=\frac{5Y_{2}\left(1-\omega \right)}{2{\left(\psi \left(h\right)\right)}^{2}}+\frac{Y_{1}K_{0}\left(\alpha \right)}{\varphi \left(k\right)\Gamma \left(2-\alpha \right)},\\ Z_{1}=Z_{5}=\frac{-Y_{2}\omega }{{\left(12\psi \left(h\right)\right)}^{2}},Z_{2}=Z_{4}=\frac{4Y_{2}\omega }{{\left(3\psi \left(h\right)\right)}^{2}},\\ Z_{3}=Y_{3}+Y_{1}\left(\frac{K_{1}\left(\alpha \right)}{\varphi {\left(k\right)}^{\left(1-\alpha \right)}}-\frac{K_{0}\left(\alpha \right)}{\varphi \left(k\right)\Gamma \left(2-\alpha \right)}\right)-\frac{5Y_{2}\omega }{{\left(2\psi \left(h\right)\right)}^{2}},\\ f=\frac{K_{0}\left(\alpha \right){\left(k\right)}^{1-\alpha }}{\varphi \left(k\right)\Gamma \left(2-\alpha \right)},s=\frac{K_{1}\left(\alpha \right)}{{\left(\psi \left(k\right)\right)}^{1-\alpha }}\text{ and }z_{k}=\left({\left(k+1\right)}^{\left(1-\alpha \right)}-k^{\left(1-\alpha \right)}\right), \end{array}$$

are the constants, where *k*=1,2,…, *m* − 1. Applying the von Neumann stability analysis by assuming that *X*_*i*_^*j*^=*χ*^*j*^*e*^*niqk*^ into system (33), where $n=\sqrt{-1}$ and *q* ∈ *R*, divide the deduced equation by *χ*^*j*^*e*^*niqk*^ and put every *χ*^*j*+1^/*χ*^*j*^=*η*. By using the Euler formulas *e*^*nϑ*^=cos(*ϑ*)+*n*sin(*ϑ*) and *e*^−*nϑ*^=cos(*ϑ*) − *n*sin(*ϑ*) and making some necessary arrangements, we will have that

(35) $$\begin{array}{c} \eta =\frac{2Z_{1}\cos\left(2qk\right)+2Z_{2}\cos\left(qk\right)+Z_{3}-f\sum_{k=1}^{m-1}\chi^{-k}z_{k}+s\sum_{k=1}^{m-1}W_{k}^{\left(1-\alpha \right)}\chi^{-k}\;}{2A_{1}\cos\left(2qk\right)+2A_{2}\cos\left(qk\right)+A_{3}-f\sum_{k=1}^{m-1}\chi^{-k}z_{k}}. \end{array}$$

The mode will be stable as long as ‖*η*‖ ≤ 1.

## 7. Results and Discussion

To clarify the performance of the proposed method for solving the suggested model, we will study the effects of various flow parameters (*α*, *ϕ*, *γ*, *M*, *K*, *Pr*, and *Gr*) that are distinguished in multifigures identifying the temperature and velocity profiles for blood. The influences of all the above parameters are displayed for human blood (SWCNTs and MWCNTs); the Pr is taken 21 and 25, respectively.

**Figure 1:**
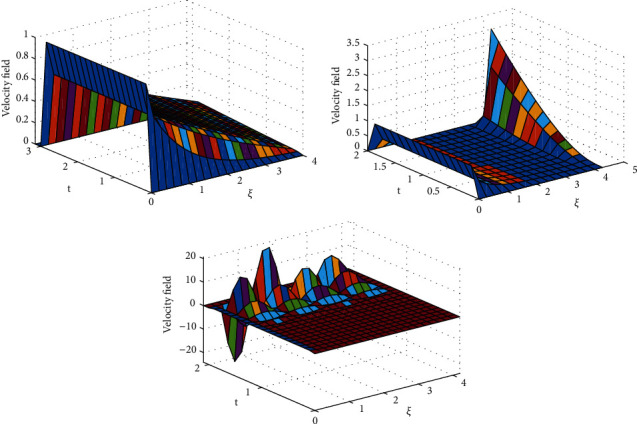
Behavior of the stable and unstable solutions for the velocity field of CNTs using the WANSCFDM.

**Figure 2:**
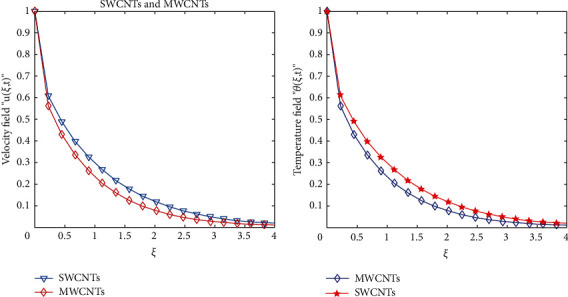
Velocity and temperature of SWCNTs and MWCNTs for human blood when *t* = 0.3, *Pr*=21, *Gr*=0.7, *M*=4, *K*=1, *γ*=0.1, *α*=0.5, *ω*=0, and *ϕ*=0.5.

**Figure 3:**
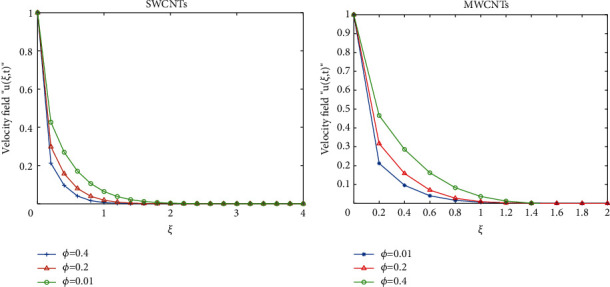
Velocity distribution of SWCNTs and MWCNTs when *t* = 1, *Pr*=25, *Gr*=0, *M*=0.5, *K*=1, *γ*=1, *α*=0.5, and *ω*=0.

**Figure 4:**
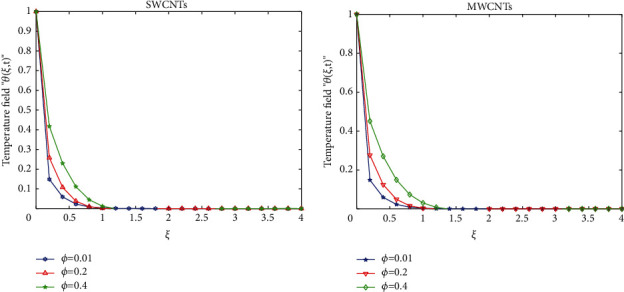
Temperature distribution of SWCNTs and MWCNTs when *t* = 1, *Pr*=25, *Gr*=0, *M*=0.5, *K*=1, *α*=0.5, *ω*=0, and *γ*=1.

**Figure 5:**
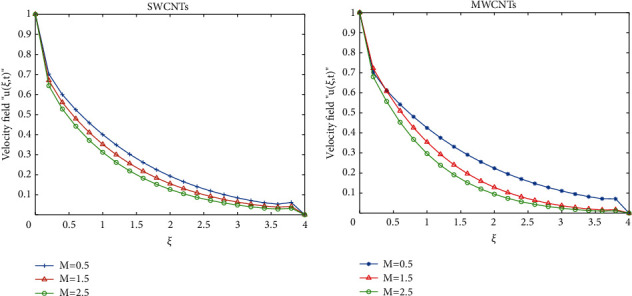
Velocity distribution of SWCNTs and MWCNTs when *t* = 0.6, *Pr*=21, *Gr*=0.7, *ϕ*=0.5, *K*=1, *α*=0.5, *ω*=0, and *γ*=0.1.

**Figure 6:**
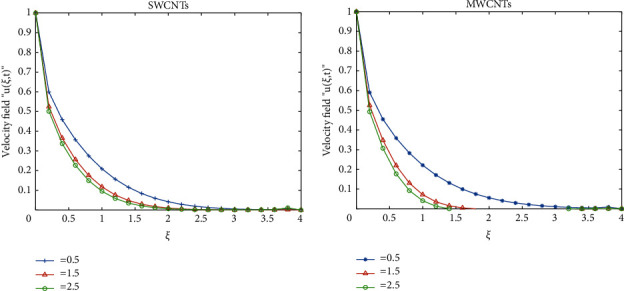
Velocity distribution of SWCNTs and MWCNTs when *t* = 0.6, *Pr*=25, *Gr*=0, *ϕ*=0.5, *K*=2, *α*=0.5, *ω*=0, and *M*=0.5.

**Figure 7:**
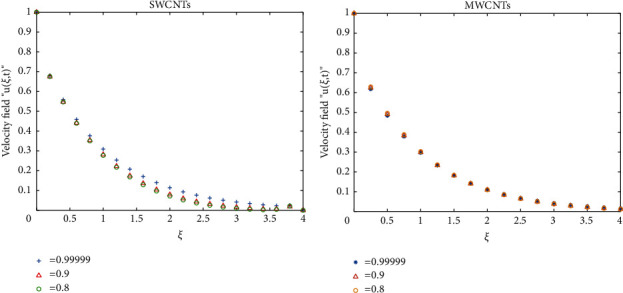
Velocity distribution at a different value of *α* when *t* = 0.6, *Pr*=21, *Gr*=0.7, *ϕ*=0.5, *K* = 1, *ω*=0, and *M* = 4.

**Figure 8:**
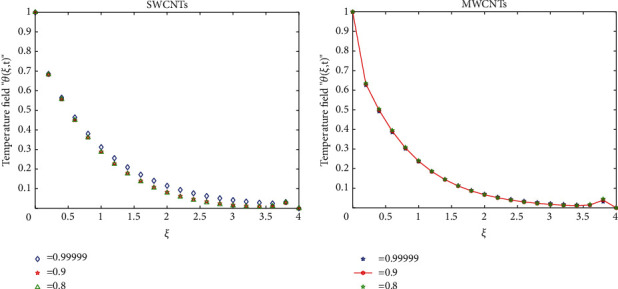
Temperature distribution at a different value of *α* when *t* = 1, *Pr*=21, *Gr*=0.7, *ϕ*=0.5, *K* = 1, *ω*=0, and *M* = 4.

- The desirable results in Figure 1 show the behavior of the stable and unstable solutions for the velocity field (22) of CNTs using the WANSCFDM
- The results in Figure 2 can be carried out from different values for *ϕ* into temperature, and velocity profiles (22) and (23) are reported for SWCNTs and MWCNTs; it is obtained from the time-fractional type and WANSCFD schemes discussed above at *α*=0.5.
- Figures 3 and 4 show the effect of the solid volume fraction *ϕ*_*q*_, (*q*=0,0.2, 0.4), *γ*=1, *Pr*=25, *K*=1, *M*=0.5, and *Gr*=0 at *t*=0.6 on SWCNTs and MWCNTs velocity and temperature profiles when *α*=0.5. It is observed that for SWCNT and MWCNT, there is an inverse relationship between velocity profile and *ϕ*, where if *ϕ* increases, the velocity of nanofluids decreases, where the changes in the velocity of nanofluids play an important part in the procedures comprising heating and cooling.
- Figure 5 shows the actions of the magnetic number on the velocity for both single wall and multiwall CNT, where it is a nondimensional number of the constant magnetic field that is the cause of Lorentz force that obverses the nanofluid velocity and resists motion, with the increasing *M* and the velocity decreasing in both cases.
- Figure 6 shows the effect of *γ* on the velocity profile, where increasing in *γ* causes the decrease in the movement of CNTs because of the lessening in the density of the momentum frontier layers. It is spotted that there is a reverse relation between *γ* and velocity for SWCNTs and MWCNTs, where *γ* increases and velocity decreases.
- Figures 7 and 8 show the numerical solutions at different values of *α*, where the temperature and velocity in SWCNTs and MWCNTs are disadvantages for increasing in values of *α* of blood
- Table 2 presents the errors (the error = the exact solution−the approximate solution) and CPU time when using the WANSCFDM and WASCFDM for SWCNT at *α*=0.8 and *γ*=1 and at a small value of *ϕ*=0.01
- Table 3 presents the errors when using the WANSCFDM and WANSFDM in case of MWCNT at *α*=0.8 and *γ*=1 and at a small value of *ϕ*=0.01
- Tables 4 and 5 provide the numerical results for the WASCFDM and WANSCFDM at different values of *ω*, such that sometimes in using the WASCFDM, we get best results from the Crank-Nicholson fractional scheme at *ω*=0.5, but for the WANSCFD method, we get the best results from the implicit fractional scheme at *ω*=0. The functions of time and space difference are as follows: *φ*(*k*)=0.001(1 − exp(−*k*)),  *ψ*(*h*)=0.5(sinh(*h*/2)).
- Table 6 provides the maximum error between the numerical solution obtained using the WANSCFDM and the exact solution using different values of N, *M* with *ω*=0 and provides the convergence order of our scheme. Observed orders of convergence for *t* component is computed using log(*eM*/*e*2*M*)/log(2), where eM and e2M are the maximum errors when the problems are solved with *M* and 2M grid points [26].

## 8. Conclusions

The proportional Caputo hybrid operator is used to fractionalize the model of the nanofluid flow of human blood CNTs over a vertical plate. The effects of the magnetic area and the porosity medium are taken into consideration. The numerical results for temperature and velocity fields are calculated by the method of WANSCFD. Numerical results are presented in diverse graphs and mentioned with physical reasonings, and all computations had been run with the use of Matlab programming. The main findings extracted are as follows [27–29]:

- The velocity of nanofluid decreases with the increase in *ϕ*, magnetic parameter, and permeability parameter
- There is an inverse relationship between the volume fraction parameter, magnetic parameter, and Casson fluid parameter
- The Casson nanofluid flow has the same influence on temperature and velocity profiles for both single and multiwalled CNTs
- CPC fractional derivative model can be qualified to solve the biological properties than the integer order model
- The numerical methods, highly accurate WANSFD, WASCFD, and WANSCFD, are used to study the presented model as shown in tables, so we can conclude by comparative results that the WANSCFDM was more accurate.
- The stability analysis of the proposed WANSCFDM is construed by a kind of the standard John von Neumann stability analysis technique
- The numerical solutions in this study are in good agreement with the exact solutions

## Figures and Tables

**Table 1:** Parameters are mentioned in the model.

Parameter	Description
*u*	Dimensionless velocity
*θ*	Dimensionless temperature
*t*	Dimensionless time
*ξ*	Dimensionless special variable
*ϕ*	Solid volume fraction of the nanofluid (0 ≤ *ϕ* < 1)
*v*_*nf*_	Constant kinematic viscosity
*Q*	Frequency of oscillation of the plate
*U*_0_	The characteristics velocity
*H*(*t*)	The unit step function
*γ*	Casson fluid parameter
*M*	Magnetic parameter
*K*	Permeability parameter
*Pr*	Prandtl number
*Gr*	Grashof number
*ρ*_*f*_	Density of the based fluid
*ρ*_*c*_	Density of the solid nanoparticle CNTs
(*C*_*p*_)_*f*_	Heat capacitance of the based fluid
(*C*_*p*_)_*c*_	Heat capacitance of the solid nanoparticles CNTs
*k*_*nf*_	Thermal conductivity of the nanofluids
*k*_*f*_	Thermal conductivity of the based fluid
*k*_*c*_	Thermal conductivity of the solid nanoparticles
*σ*_*f*_	The electrical conductivity of the based fluid
*β*_*f*_	Thermal expansion coefficient of the based fluid
*β*_*c*_	Thermal expansion coefficient of the solid nanoparticles CNTs

**Table 2:** The errors and CPU time for the WANSCFDM and WANSFDM for SWCNTs when *ω*=0, *ϕ*=0.01, *Gr*=0, *Pr*=25, *t*=0.9, *K* = 1, *M* = 0.5, *γ*=1, and *α*=0.8.

*ξ*	WANSCFDM	CPU time for WANSCFDM (s)	WASCFDM	CPU time for WASCFDM (s)
2	2.197 × 10^−5^	117.42	9.377 × 10^−3^	141.92
2.4	5.614 × 10^−6^	125.24	5.095 × 10^−3^	151.38
2.8	1.161 × 10^−6^	133.07	2.733 × 10^−3^	160.84
3.2	2.193 × 10^−7^	140.90	1.397 × 10^−3^	170.30
3.6	3.3422 × 10^−8^	148.73	2.211 × 10^−4^	179.76
4	6.853 × 10^−16^	156.56	1.971 × 10^−14^	189.22

**Table 3:** The errors of the WANSCFDM and WANSFDM for MWCNTs when *ω*=0, *ϕ*=0.01, *Gr*=0, *Pr*=25, *t*=0.9, *K* = 1, *M* = 0.5, *γ*=1, and *α*=0.8.

*ξ*	WANSCFDM	WANSFDM
2	3.4250 × 10^−5^	2.5440 × 10^−2^
2.4	1.1162 × 10^−5^	1.6140 × 10^−2^
2.8	2.5435 × 10^−6^	9.8587 × 10^−3^
3.2	5.0052 × 10^−7^	5.5662 × 10^−3^
3.6	9.0993 × 10^−8^	2.4624 × 10^−3^
4	7.2880 × 10^−16^	1.2795 × 10^−12^

**Table 4:** The error of the WASCFD method when *ϕ*=0.02, *Gr*=0, *Pr*=21, *t*=0.6, *K* = 2, *M* = 0.5, *γ*=1, and *α*=0.9.

*ξ*	*ω*=0	*ω*=0.5	*ω*=1
2	2.9944 × 10^−3^	3.0617 × 10^−3^	7.3117 × 10^−2^
2.4	1.8007 × 10^−3^	1.8345 × 10^−3^	2.9043 × 10^−2^
2.8	9.9935 × 10^−4^	1.0104 × 10^−3^	2.4336 × 10^−2^
3.2	5.1208 × 10^−4^	5.0655 × 10^−4^	1.5660 × 10^−2^
3.6	2.1095 × 10^−4^	2.0303 × 10^−4^	3.0798 × 10^−2^
4	2.8200 × 10^−7^	2.8200 × 10^−7^	1.3834 × 10^−5^

**Table 5:** The error of the WANSCFD method when *ϕ*=0.02, *Gr*=0, *Pr*=25, *t*=1, *K* = 1, *M* = 0.5, *γ*=1, and *α*=0.9.

*ξ*	*ω*=0	*ω*=0.5	*ω*=1
1.4	5.9641 × 10^−5^	4.4824 × 10^−6^	6.8929 × 10^−3^
1.8	7.8977 × 10^−5^	8.9393 × 10^−5^	2.2885 × 10^−3^
2.2	2.9028 × 10^−5^	3.0682 × 10^−5^	7.1471 × 10^−3^
2.6	7.1437 × 10^−6^	7.3531 × 10^−6^	2.1694 × 10^−4^
3	1.4854 × 10^−6^	1.5038 × 10^−6^	6.5119 × 10^−5^
3.4	2.8676 × 10^−7^	2.8799 × 10^−7^	1.9461 × 10^−5^
3.8	4.4572 × 10^−8^	4.4688 × 10^−8^	5.7848 × 10^−6^
4.2	1.5867 × 10^−17^	1.4310 × 10^−17^	1.7049 × 10^−6^

**Table 6:** The *e* maximum errors and the convergence order for SWCNTs using WANSCFD at *ω*=0.

*ξ*	*N* = 20, *M* = 20	*N* = 20, *M* = 40	Order
0.6	2.32 09 ×10^−3^	6.4443 ×10^−4^	1.84
1.2	8.8077 ×10^−3^	3.8068 ×10^−3^	1.21
1.8	1.2862 ×10^−2^	9.5632 ×10^−3^	0.42
2.4	1.5655 ×10^−2^	7.5276 ×10^−3^	1.05
3	0.18286	4.2939 ×10^−2^	2.09

## Data Availability

No data were used to support this study.

## Supplementary Materials

Supplementary Materials Matlab programme for the article NO. 3520833, “A Fourth-Order Compact Finite Difference Schemes for Solving Time Fractional Carbon Nanotubes Model.”.
