# Supplementary material for: A Fourth-Order Compact Finite Difference Scheme for Solving the Time Fractional Carbon Nanotubes Model
Source: ScientificWorldJournal. 2022 Mar 2;2022:1426837. doi: 10.1155/2022/1426837 (PMC8906970; doi:10.1155/2022/1426837)
Supplement: Supplementary Materials — Matlab programme for the article NO. 3520833, “A Fourth-Order Compact Finite Difference Schemes for Solving Time Fractional Carbon Nanotubes Model.”. [file 1426837.f1.pdf]

Matlab programme for the article NO. 3520833, "A Fourth-Order Compact Finite Difference Schemes for Solving Time Fractional Carbon Nanotubes Model."

```
%-----------------------------------------------------
%  System Ex.1m-thermoelast
clear,  close all;   clc;
global  n m sigma dy dt y1 t1 alpha1;
n=20;   m=20;
%sigma=1; %Implict
sigma=0; %Implict
%sigma=1/2; %crank
y1=0;   yend=4;   t1=0.0001;   tend=2;
dy=(yend-y1)/(n);
dt=(tend-t1)/(m);
u0 = zeros((n+2),(m+2));
t(1)=t1;   y(1)=y1;     alpha1=0.99;
%M=0.5;   gma=0.5;  Q=0.5;   Gr=0;  Pr=25;  K=2;
M=1;   gma=0.05;  Q=0.02;   Gr=0.7;  Pr=25;  K=2;
%M=4;   gma=0.1;  Q=0.5;   Gr=0.7;  Pr=21;  K=1;
%M=5;   gma=1;  Q=0.1;   Gr=0;  Pr=21;  K=2;
%M=0.5;   gma=1;  Q=0.7;   Gr=0.1;  Pr=25;  K=1;
%%%%%%%%%%
Ro_f=1053;    Ro_m=1600;
sgm_f=0.8;    sgm_m=1.9*10^(-4);
Beta_f=0.18*10^(5);  Beta_m=44*10^(5);
CP_f=3594;    CP_m=796;
K_f=0.492;    K_m=3000;
%%%%%%%%
Ro_nf=(1-Q)*Ro_f + Q*Ro_m;
MEO_nf = M/(1-Q)^(2.5);
sgm_nf = (1+((3*((sgm_m/sgm_f)-1)*Q)/((((sgm_m/sgm_f)+2)-
((sgm_m/sgm_f)-1))*Q))))*sgm_f;
Beta_nf = ((1-Q)*(Ro_f*Beta_f)+Q*(Ro_m*Beta_m))/Ro_nf;
RoCP_nf = (1-Q)*(Ro_f*CP_f)+Q*(Ro_m*CP_m);
K_nf=(((1-Q)+2*Q*(K_m/(K_m-K_f))*log((K_m+K_f)/(2*K_f)))/((1-
Q)+2*Q*(K_f/(K_m-K_f))*log((K_m+K_f)/(2*K_f))))*K_f;
%%%%%%%%
Q1=((1-Q)^2.5)*((1-Q)+Q*(Ro_m/Ro_f));
Q2=1+(1-Q)*((3*Q*((sgm_m/sgm_f)-1))/(((sgm_m/sgm_f)+2)-
Q*((sgm_m/sgm_f)-1)))+Q*(Ro_m/Ro_f);
Q3=((1-Q)+Q*((Ro_m*Beta_m)/(Ro_f*Beta_f)))/((1-Q)+Q*(Ro_m/Ro_f));
Q4=(1-Q)+Q*((Ro_m*CP_m)/(Ro_f*CP_f));
Q5=(K_nf/K_f);
%%%%%%%%
A = (1/Q1)*(1+(1/gma));
B = (M/Q2)+(Q1/K);
C = Q3*Gr;
%%%%%%%%
a0=(gma/(1+gma));
a1=a0*Q1;
b0=((Pr*Q3)/Q5);
b1=((a1*Gr*Q1*Q2)/(b0-1));
L=(M/Q2)+(Q1/K);
%%%%%%%%%
k0=(alpha1)*((tend)^(1-alpha1));
k1=(1-alpha1)*(tend^(alpha1));
%%%%%%%%%
for j=1:m
   t(j+1)=t(j)+dt;
end
```

```
for i=1:n
    y(i+1)=y(i)+dy;
end
u = fsolve(@MWCNTvelocity_Cacom3520833,u0);
%boundary  and initial u
for mm=1:m+1    %u(1,mm)=1
    u(1,mm)=((1/2)*(exp(-
1*((a1*L)^(1/2)))*erfc((1/2)*((a1/mm)^(1/2))-((L*mm)^(1/2)))+...

exp(1*((a1*L)^(1/2)))*erfc((1/2)*((a1/mm)^(1/2))+((L*mm)^(1/2))))+...
                   b1*((exp(-
1*((a1*L)^(1/2)))*erfc((1/2)*((a1/mm)^(1/2)))-
((L*mm)^(1/2)))*((mm/2)-(1/4)*((a1/L)^(1/2)))+...

(exp(1*((a1*L)^(1/2)))*erfc((1/2)*((a1/mm)^(1/2)))+((L*mm)^(1/2)))*((
mm/2)+(1/4)*((a1/L)^(1/2))))-...

b1*((mm+((b0*(1^2))/2))*erfc((1/2)*((b0/mm)^(1/2)))-
(1*((b0)^(1/2))*((mm/pi)^(1/2))*exp(-((b0*(1^2))/(4*mm))))));
   % u(n+1,mm)=0;
end
for i=1:n+1 , u(i,1)=0;  u(i,mm)=0;  end
%%%%%
u;
%%  exact
for j=1:m+1
for i=1:n+1
 approxcomplex(i,j)=u(i,j);
 exact(i,j)=(1/2)*(exp(-i*((a1*L)^(1/2)))*erfc((i/2)*((a1/j)^(1/2))-
((L*j)^(1/2)))+...
exp(i*((a1*L)^(1/2)))*erfc((i/2)*((a1/j)^(1/2))+((L*j)^(1/2))))+...
b1*((exp(-i*((a1*L)^(1/2)))*erfc((i/2)*((a1/j)^(1/2)))-
((L*j)^(1/2)))*((j/2)-(i/4)*((a1/L)^(1/2)))+...
(exp(i*((a1*L)^(1/2)))*erfc((i/2)*((a1/j)^(1/2)))+((L*j)^(1/2)))*((j/
2)+(i/4)*((a1/L)^(1/2))))-...
 b1*((j+((b0*(i^2))/2))*erfc((i/2)*((b0/j)^(1/2)))-
i*((b0)^(1/2))*((j/pi)^(1/2))*exp(-(b0*(i^2))/(4*j)));

 error1(i,j)=abs(exact(i,j)-u(i,j));
end
end
 approxcomplex;

 exact;

figure(1)
surf(y,t,(approxcomplex(:,:))')
figure(2)
plot(y,(approxcomplex(:,20))')

  figure(3)
  plot(y,(exact(:,20))')

figure(3)
  plot(y,(exact(:,10)),'*')
  hold on
  plot(y,(approxcomplex(:,10)),'+')  %alpha=0.99999
 ylabel('Velocity filed "u(\xi,t)" ')
```

```
 function zz = MWCNTvelocity_Cacom3520833(u)
global  n m sigma dy dt y1 tt yy t1 alpha1;
%%%%%%%%
y1=0;   yend=4;   t1=0.0001;   tend=2;   w1(1)=1;
dy=(yend-y1)/(n);
dt=(tend-t1)/(m);
%%%%%%%%
%M=0.5;   gma=0.5;  Q=0.5;   Gr=0;  Pr=25;  K=2;
%M=0.5;   gma=0.1;  Q=0.5;   Gr=0.7;  Pr=21;  K=2;
%M=4;   gma=0.1;  Q=0.5;   Gr=0.7;  Pr=21;  K=1;
%M=5;   gma=1;  Q=0.1;   Gr=0;  Pr=21;  K=2;
%M=0.5;   gma=1;  Q=0.7;   Gr=0.1;  Pr=25;  K=1;
M=1;   gma=0.05;  Q=0.02;   Gr=0.7;  Pr=25;  K=2;

%%%%%%%%%%%%%%
Ro_f=1053;    Ro_m=1600;
sgm_f=0.8;    sgm_m=1.9*10^(-4);
Beta_f=0.18*10^(5);  Beta_m=44*10^(5);
CP_f=3594;    CP_m=796;
K_f=0.492;    K_m=3000;
%%%%%%%%
Ro_nf=(1-Q)*Ro_f + Q*Ro_m;
MEO_nf = M/(1-Q)^(2.5);
sgm_nf = (1+((3*((sgm_m/sgm_f)-1)*Q)/((((sgm_m/sgm_f)+2)-
((sgm_m/sgm_f)-1))*Q)))*sgm_f;
Beta_nf = ((1-Q)*(Ro_f*Beta_f)+Q*(Ro_m*Beta_m))/Ro_nf;
RoCP_nf = (1-Q)*(Ro_f*CP_f)+Q*(Ro_m*CP_m);
K_nf=(((1-Q)+2*Q*(K_m/(K_m-K_f))*log((K_m+K_f)/(2*K_f)))/((1-
Q)+2*Q*(K_f/(K_m-K_f))*log((K_m+K_f)/(2*K_f))))*K_f;
%%%%%%%%
Q1=((1-Q)^2.5)*((1-Q)+Q*(Ro_m/Ro_f));
Q2=1+(1-Q)*((3*Q*((sgm_m/sgm_f)-1))/(((sgm_m/sgm_f)+2)-
Q*((sgm_m/sgm_f)-1)))+Q*(Ro_m/Ro_f);
Q3=((1-Q)+Q*((Ro_m*Beta_m)/(Ro_f*Beta_f)))/((1-Q)+Q*(Ro_m/Ro_f));
Q4=(1-Q)+Q*((Ro_m*CP_m)/(Ro_f*CP_f));
Q5=(K_nf/K_f);
%%%%%%%%
A = (1/Q1)*(1+(1/gma));
B = (M/Q2)+(Q1/K);
C = Q3*Gr;
%%%%%%%%
a0=(gma/(1+gma));
a1=a0*Q1;
b0=((Pr*Q3)/Q5);
b1=((a1*Gr*Q1*Q2)/(b0-1));
L=(M/Q2)+(Q1/K);
%%%%%%%%%
k0=(alpha1)*((tend)^(1-alpha1));
k1=(1-alpha1)*(tend^(alpha1));
%%%%%%%%%
tt=0.001*(1-exp(-dt));
yy=0.5*sinh(dy/2);
%%%%%%%%
 %boundary
for mm=1:m+1
v(1,mm)=1;    v(n+1,mm)=0;
end
%initial
for i=1:n+1
```

```
v(i,1)=0;         v(i,mm)=0;
v(i,m+1)=1;
end
L1=(1*((tt^(1-alpha1))^(-1)));
%L1=(1*((dt^(1-alpha1))^(-1)));
for k=2:mm
end
%%%%%%%%%%
 for mm=2:m
 for i=3:n
 sum_u=0;
 sum1_u=0;
         for  k=0:mm-2
 sum_u=sum_u+(u(i,mm+1-k)-u(i,mm-k))*((k+1)^(1-alpha1)-(k)^(1-
alpha1))+...
     (u(i,mm+1-(mm-1))-u(i,mm-(mm-1)))*((mm-1+1)^(1-alpha1)-(mm-
1)^(1-alpha1));
         end
         for k=2:mm
  w1(k)=(1-((2-alpha1).*(k.^(-1))))*(w1(k-1));
  sum1_u = sum1_u + (w1(k)*u(i,mm+1-k));
         end
zu(i,mm+1)=(k1*(((u(i,mm+1)-
u(i,mm))/tt)+(L1*u(i,mm+1))+(L1*sum1_u))+k0*(sum_u*((tt)^(-
alpha1)/gamma(2-alpha1))))-A*(sigma)*((-
(1/12)*u(i+2,mm)+(4/3)*u(i+1,mm)-(5/2)*u(i,mm)+(4/3)*u(i-1,mm)-
(1/12)*u(i-2,mm))/(yy^2))-A*(1-sigma)*((-
(1/12)*u(i+2,mm+1)+(4/3)*u(i+1,mm+1)-(5/2)*u(i,mm+1)+(4/3)*u(i-
1,mm+1)-(1/12)*u(i-2,mm+1))/(yy^2))+(B-1)*u(i,mm)-C*v(i,mm);
%zu(i,mm+1)=(k1*(((u(i,mm+1)-
u(i,mm))/dt)+(L1*u(i,mm+1))+(L1*sum1_u))+k0*(sum_u*((dt)^(-
alpha1)/gamma(2-alpha1))))-A*(sigma)*((-
(1/12)*u(i+2,mm)+(4/3)*u(i+1,mm)-(5/2)*u(i,mm)+(4/3)*u(i-1,mm)-
(1/12)*u(i-2,mm))/(dy^2))-A*(1-sigma)*((-
(1/12)*u(i+2,mm+1)+(4/3)*u(i+1,mm+1)-(5/2)*u(i,mm+1)+(4/3)*u(i-
1,mm+1)-(1/12)*u(i-2,mm+1))/(dy^2))+(B-1)*u(i,mm)-C*v(i,mm);
%zu(i,mm+1)=k1*(((u(i,mm+1)-
u(i,mm))/tt)+(L1*u(i,mm+1))+(L1*sum1_u))+k0*(sum_u*((tt)^(-
alpha1)/gamma(2-alpha1)))-A*(sigma)*((-
(u(i+2,mm)/12)+(4*u(i+1,mm)/3)-(5*u(i,mm)/2)+(4*u(i-1,mm)/3)-(u(i-
2,mm)/12))/(yy^2))-A*(1-sigma)*((-(u(i+2,mm+1)/12)+(4*u(i+1,mm+1)/3)-
(5*u(i,mm+1)/2)+(4*u(i-1,mm+1)/3)-(u(i-2,mm+1)/12))/(yy^2))+(B-
1)*u(i,mm)-C*v(i,mm);
%%%%%%%%%%%%
v(1,mm)=erfc((1/2)*(b0/mm)^(1/2));
v(1,1)=erfc((1/2)*(b0/1)^(1/2));
v(n+1,mm)=erfc(((n+1)/2)*(b0/mm)^(1/2));
v(n+1,1)=erfc(((n+1)/2)*(b0/1)^(1/2));
v(i,1)=erfc((i/2)*(b0/1)^(1/2));
v(n+1,1)=erfc(((n+1)/2)*(b0/1)^(1/2));
v(1,2)=erfc((1/2)*(b0/2)^(1/2));
v(i,2)=erfc((i/2)*(b0/2)^(1/2));
v(1,m+1)=erfc((1/2)*(b0/(m+1))^(1/2));
v(n+1,m+1)=erfc(((n+1)/2)*(b0/(m+1))^(1/2));
v(i,mm)=erfc((i/2)*(b0/mm)^(1/2));
% %conditions of u
  zu(1,mm)=u(1,mm)-((1/2)*(exp(-
1*((a1*L)^(1/2)))*erfc((1/2)*((a1/mm)^(1/2))-((L*mm)^(1/2)))+...

exp(1*((a1*L)^(1/2)))*erfc((1/2)*((a1/mm)^(1/2))+((L*mm)^(1/2))))+...
```

```
                         b1*((exp(-
1*((a1*L)^(1/2)))*erfc((1/2)*((a1/mm)^(1/2)))-
((L*mm)^(1/2)))*((mm/2)-(1/4)*((a1/L)^(1/2)))+...

(exp(1*((a1*L)^(1/2)))*erfc((1/2)*((a1/mm)^(1/2)))+((L*mm)^(1/2)))*((
mm/2)+(1/4)*((a1/L)^(1/2)))))-...

b1*((mm+((b0*(1^2))/2))*erfc((1/2)*((b0/mm)^(1/2)))-
(1*((b0)^(1/2))*((mm/pi)^(1/2))*exp(-((b0*(1^2))/(4*mm))))));

    zu(2,mm)=u(2,mm)-((1/2)*(exp(-
2*((a1*L)^(1/2)))*erfc((2/2)*((a1/mm)^(1/2))-((L*mm)^(1/2)))+...

exp(2*((a1*L)^(1/2)))*erfc((2/2)*((a1/mm)^(1/2))+((L*mm)^(1/2))))+...
                         b1*((exp(-
2*((a1*L)^(1/2)))*erfc((2/2)*((a1/mm)^(1/2)))-
((L*mm)^(1/2)))*((mm/2)-(2/4)*((a1/L)^(1/2)))+...

(exp(2*((a1*L)^(1/2)))*erfc((2/2)*((a1/mm)^(1/2)))+((L*mm)^(1/2)))*((
mm/2)+(2/4)*((a1/L)^(1/2)))))-...

b1*((mm+((b0*(2^2))/2))*erfc((2/2)*((b0/mm)^(1/2)))-
(2*((b0)^(1/2))*((mm/pi)^(1/2))*exp(-((b0*(2^2))/(4*mm))))));
% %

   zu(1,1)=u(1,1)-((1/2)*(exp(-
1*((a1*L)^(1/2)))*erfc((1/2)*((a1/1)^(1/2))-((L*1)^(1/2)))+...

exp(1*((a1*L)^(1/2)))*erfc((1/2)*((a1/1)^(1/2))+((L*1)^(1/2))))+...
                         b1*((exp(-
1*((a1*L)^(1/2)))*erfc((1/2)*((a1/1)^(1/2)))-((L*1)^(1/2)))*((1/2)-
(1/4)*((a1/L)^(1/2)))+...

(exp(1*((a1*L)^(1/2)))*erfc((1/2)*((a1/1)^(1/2)))+((L*1)^(1/2)))*((1/
2)+(1/4)*((a1/L)^(1/2)))))-...
                         b1*((1+((b0*(1^2))/2))*erfc((1/2)*((b0/1)^(1/2)))-
(1*((b0)^(1/2))*((1/pi)^(1/2))*exp(-((b0*(1^2))/(4*1))))));
    zu(2,2)=u(2,2)-((1/2)*(exp(-
(2)*((a1*L)^(1/2)))*erfc(((2)/2)*((a1/2)^(1/2))-((L*2)^(1/2)))+...

exp((2)*((a1*L)^(1/2)))*erfc(((2)/2)*((a1/2)^(1/2))+((L*2)^(1/2))))+.
..
                         b1*((exp(-
(2)*((a1*L)^(1/2)))*erfc(((2)/2)*((a1/2)^(1/2)))-
((L*2)^(1/2)))*((2/2)-((2)/4)*((a1/L)^(1/2)))+...

(exp((2)*((a1*L)^(1/2)))*erfc(((2)/2)*((a1/2)^(1/2)))+((L*2)^(1/2)))*
((2/2)+((2)/4)*((a1/L)^(1/2)))))-...

b1*((2+((b0*((2)^2))/2))*erfc(((2)/2)*((b0/2)^(1/2)))-
(2)*((b0)^(1/2))*((2/pi)^(1/2))*exp(-(b0*((2)^2))/(4*2))));
%
   zu(n+2,mm)=u(n+2,mm)-((1/2)*(exp(-
(n+2)*((a1*L)^(1/2)))*erfc(((n+2)/2)*((a1/mm)^(1/2))-
((L*mm)^(1/2)))+...

exp((n+2)*((a1*L)^(1/2)))*erfc(((n+2)/2)*((a1/mm)^(1/2))+((L*mm)^(1/2
))))+...
                         b1*((exp(-
(n+2)*((a1*L)^(1/2)))*erfc(((n+2)/2)*((a1/mm)^(1/2)))-
((L*mm)^(1/2)))*((mm/2)-((n+2)/4)*((a1/L)^(1/2)))+...
```

```
(exp((n+2)*((a1*L)^(1/2)))*erfc(((n+2)/2)*((a1/mm)^(1/2)))+((L*mm)^(1
/2)))*((mm/2)+((n+2)/4)*((a1/L)^(1/2))))-...

b1*((mm+((b0*((n+2)^2))/2))*erfc(((n+2)/2)*((b0/mm)^(1/2)))-
(n+2)*((b0)^(1/2))*((mm/pi)^(1/2))*exp(-(b0*((n+2)^2))/(4*mm))));
%
   zu(n+1,mm)=u(n+1,mm)-((1/2)*(exp(-
(n+1)*((a1*L)^(1/2)))*erfc(((n+1)/2)*((a1/mm)^(1/2))-
((L*mm)^(1/2)))+...

exp((n+1)*((a1*L)^(1/2)))*erfc(((n+1)/2)*((a1/mm)^(1/2))+((L*mm)^(1/2
))))+...
                   b1*((exp(-
(n+1)*((a1*L)^(1/2)))*erfc(((n+1)/2)*((a1/mm)^(1/2)))-
((L*mm)^(1/2)))*((mm/2)-((n+1)/4)*((a1/L)^(1/2)))+...

(exp((n+1)*((a1*L)^(1/2)))*erfc(((n+1)/2)*((a1/mm)^(1/2)))+((L*mm)^(1
/2)))*((mm/2)+((n+1)/4)*((a1/L)^(1/2))))-...

b1*((mm+((b0*((n+1)^2))/2))*erfc(((n+1)/2)*((b0/mm)^(1/2)))-
(n+1)*((b0)^(1/2))*((mm/pi)^(1/2))*exp(-(b0*((n+1)^2))/(4*mm))));
%
   zu(n+1,1)=u(n+1,1)-((1/2)*(exp(-
(n+1)*((a1*L)^(1/2)))*erfc(((n+1)/2)*((a1/1)^(1/2))-
((L*1)^(1/2)))+...

exp((n+1)*((a1*L)^(1/2)))*erfc(((n+1)/2)*((a1/1)^(1/2))+((L*1)^(1/2))
))+...
                   b1*((exp(-
(n+1)*((a1*L)^(1/2)))*erfc(((n+1)/2)*((a1/1)^(1/2)))-
((L*1)^(1/2)))*((1/2)-((n+1)/4)*((a1/L)^(1/2)))+...

(exp((n+1)*((a1*L)^(1/2)))*erfc(((n+1)/2)*((a1/1)^(1/2)))+((L*1)^(1/2
)))*((1/2)+((n+1)/4)*((a1/L)^(1/2))))-...

b1*((1+((b0*((n+1)^2))/2))*erfc(((n+1)/2)*((b0/1)^(1/2)))-
(n+1)*((b0)^(1/2))*((1/pi)^(1/2))*exp(-(b0*((n+1)^2))/(4*1))));
zu(n+2,1)=u(n+2,1)-((1/2)*(exp(-
(n+2)*((a1*L)^(1/2)))*erfc(((n+2)/2)*((a1/1)^(1/2))-
((L*1)^(1/2)))+...

exp((n+2)*((a1*L)^(1/2)))*erfc(((n+2)/2)*((a1/1)^(1/2))+((L*1)^(1/2))
))+...
                   b1*((exp(-
(n+2)*((a1*L)^(1/2)))*erfc(((n+2)/2)*((a1/1)^(1/2)))-
((L*1)^(1/2)))*((1/2)-((n+2)/4)*((a1/L)^(1/2)))+...

(exp((n+2)*((a1*L)^(1/2)))*erfc(((n+2)/2)*((a1/1)^(1/2)))+((L*1)^(1/2
)))*((1/2)+((n+2)/4)*((a1/L)^(1/2))))-...

b1*((1+((b0*((n+2)^2))/2))*erfc(((n+2)/2)*((b0/1)^(1/2)))-
(n+2)*((b0)^(1/2))*((1/pi)^(1/2))*exp(-(b0*((n+2)^2))/(4*1))));


   zu(i,1)=u(i,1)-((1/2)*(exp(-
i*((a1*L)^(1/2)))*erfc((i/2)*((a1/1)^(1/2))-((L*1)^(1/2)))+...

exp(i*((a1*L)^(1/2)))*erfc((i/2)*((a1/1)^(1/2))+((L*1)^(1/2))))+...
                   b1*((exp(-
i*((a1*L)^(1/2)))*erfc((i/2)*((a1/1)^(1/2)))-((L*1)^(1/2)))*((1/2)-
(i/4)*((a1/L)^(1/2)))+...
```

```
(exp(i*((a1*L)^(1/2)))*erfc((i/2)*((a1/1)^(1/2)))+((L*1)^(1/2)))*((1/
2)+(i/4)*((a1/L)^(1/2))))-...
                      b1*((1+((b0*(i^2))/2))*erfc((i/2)*((b0/1)^(1/2)))-
i*((b0)^(1/2))*((1/pi)^(1/2))*exp(-(b0*(i^2))/(4*1))));

  zu(1,2)=u(1,2)-((1/2)*(exp(-
1*((a1*L)^(1/2)))*erfc((1/2)*((a1/2)^(1/2))-((L*2)^(1/2)))+...

exp(1*((a1*L)^(1/2)))*erfc((1/2)*((a1/2)^(1/2))+((L*2)^(1/2))))+...
                      b1*((exp(-
1*((a1*L)^(1/2)))*erfc((1/2)*((a1/2)^(1/2)))-((L*2)^(1/2)))*((2/2)-
(1/4)*((a1/L)^(1/2)))+...

(exp(1*((a1*L)^(1/2)))*erfc((1/2)*((a1/2)^(1/2)))+((L*2)^(1/2)))*((2/
2)+(1/4)*((a1/L)^(1/2))))-...
                      b1*((2+((b0*(1^2))/2))*erfc((1/2)*((b0/2)^(1/2)))-
1*((b0)^(1/2))*((2/pi)^(1/2))*exp(-(b0*(1^2))/(4*2))));

 zu(2,1)=u(2,1)-((1/2)*(exp(-
2*((a1*L)^(1/2)))*erfc((2/2)*((a1/(1))^(1/2))-((L*(1))^(1/2)))+...

exp(2*((a1*L)^(1/2)))*erfc((2/2)*((a1/(1))^(1/2))+((L*(1))^(1/2))))+.
..
                      b1*((exp(-
2*((a1*L)^(1/2)))*erfc((2/2)*((a1/(1))^(1/2)))-
((L*(1))^(1/2)))*(((1)/2)-(2/4)*((a1/L)^(1/2)))+...

(exp(2*((a1*L)^(1/2)))*erfc((2/2)*((a1/(1))^(1/2)))+((L*(1))^(1/2)))*
(((1)/2)+(2/4)*((a1/L)^(1/2))))-...

b1*(((1)+((b0*(2^2))/2))*erfc((2/2)*((b0/(1))^(1/2)))-
(2*((b0)^(1/2))*(((1)/pi)^(1/2))*exp(-((b0*(2^2))/(4*(1)))))));

   zu(i,2)=u(i,2)-((1/2)*(exp(-
i*((a1*L)^(1/2)))*erfc((i/2)*((a1/2)^(1/2))-((L*2)^(1/2)))+...

exp(i*((a1*L)^(1/2)))*erfc((i/2)*((a1/2)^(1/2))+((L*2)^(1/2))))+...
                      b1*((exp(-
i*((a1*L)^(1/2)))*erfc((i/2)*((a1/2)^(1/2)))-((L*2)^(1/2)))*((2/2)-
(i/4)*((a1/L)^(1/2)))+...

(exp(i*((a1*L)^(1/2)))*erfc((i/2)*((a1/2)^(1/2)))+((L*2)^(1/2)))*((2/
2)+(i/4)*((a1/L)^(1/2))))-...
                      b1*((2+((b0*(i^2))/2))*erfc((i/2)*((b0/2)^(1/2)))-
i*((b0)^(1/2))*((2/pi)^(1/2))*exp(-(b0*(i^2))/(4*2))));

  zu(1,m+1)=u(1,m+1)-((1/2)*(exp(-
1*((a1*L)^(1/2)))*erfc((1/2)*((a1/(m+1))^(1/2))-
((L*(m+1))^(1/2)))+...

exp(1*((a1*L)^(1/2)))*erfc((1/2)*((a1/(m+1))^(1/2))+((L*(m+1))^(1/2))
))+...
                      b1*((exp(-
1*((a1*L)^(1/2)))*erfc((1/2)*((a1/(m+1))^(1/2)))-
((L*(m+1))^(1/2)))*(((m+1)/2)-(1/4)*((a1/L)^(1/2)))+...

(exp(1*((a1*L)^(1/2)))*erfc((1/2)*((a1/(m+1))^(1/2)))+((L*(m+1))^(1/2
)))*(((m+1)/2)+(1/4)*((a1/L)^(1/2))))-...
```

```
b1*(((m+1)+((b0*(1^2))/2))*erfc((1/2)*((b0/(m+1))^(1/2)))-
(1*((b0)^(1/2))*(((m+1)/pi)^(1/2))*exp(-((b0*(1^2))/(4*(m+1)))))));

  zu(n+1,m+1)=u(n+1,m+1)-((1/2)*(exp(-
(n+1)*((a1*L)^(1/2)))*erfc(((n+1)/2)*((a1/(m+1))^(1/2))-
((L*(m+1))^(1/2)))+...

exp((n+1)*((a1*L)^(1/2)))*erfc(((n+1)/2)*((a1/(m+1))^(1/2))+((L*(m+1)
)^(1/2))))+...
                   b1*((exp(-
(n+1)*((a1*L)^(1/2)))*erfc(((n+1)/2)*((a1/(m+1))^(1/2)))-
((L*(m+1))^(1/2)))*(((m+1)/2)-((n+1)/4)*((a1/L)^(1/2)))+...

(exp((n+1)*((a1*L)^(1/2)))*erfc(((n+1)/2)*((a1/(m+1))^(1/2)))+((L*(m+
1))^(1/2)))*(((m+1)/2)+((n+1)/4)*((a1/L)^(1/2))))-...

b1*(((m+1)+((b0*((n+1)^2))/2))*erfc(((n+1)/2)*((b0/(m+1))^(1/2)))-
(n+1)*((b0)^(1/2))*(((m+1)/pi)^(1/2))*exp(-
(b0*((n+1)^2))/(4*(m+1)))));
 zu(n+2,m+1)=u(n+2,m+1)-((1/2)*(exp(-
(n+2)*((a1*L)^(1/2)))*erfc(((n+2)/2)*((a1/(m+1))^(1/2))-
((L*(m+1))^(1/2)))+...

exp((n+2)*((a1*L)^(1/2)))*erfc(((n+2)/2)*((a1/(m+1))^(1/2))+((L*(m+1)
)^(1/2))))+...
                   b1*((exp(-
(n+2)*((a1*L)^(1/2)))*erfc(((n+2)/2)*((a1/(m+1))^(1/2)))-
((L*(m+1))^(1/2)))*(((m+1)/2)-((n+2)/4)*((a1/L)^(1/2)))+...

(exp((n+2)*((a1*L)^(1/2)))*erfc(((n+2)/2)*((a1/(m+1))^(1/2)))+((L*(m+
1))^(1/2)))*(((m+1)/2)+((n+2)/4)*((a1/L)^(1/2))))-...

b1*(((m+1)+((b0*((n+2)^2))/2))*erfc(((n+2)/2)*((b0/(m+1))^(1/2)))-
(n+2)*((b0)^(1/2))*(((m+1)/pi)^(1/2))*exp(-
(b0*((n+2)^2))/(4*(m+1)))));
%

  zu(2,m+1)=u(2,m+1)-((1/2)*(exp(-
(2)*((a1*L)^(1/2)))*erfc(((2)/2)*((a1/(m+1))^(1/2))-
((L*(m+1))^(1/2)))+...

exp((2)*((a1*L)^(1/2)))*erfc(((2)/2)*((a1/(m+1))^(1/2))+((L*(m+1))^(1
/2))))+...
                   b1*((exp(-
(2)*((a1*L)^(1/2)))*erfc(((2)/2)*((a1/(m+1))^(1/2)))-
((L*(m+1))^(1/2)))*(((m+1)/2)-((2)/4)*((a1/L)^(1/2)))+...

(exp((2)*((a1*L)^(1/2)))*erfc(((2)/2)*((a1/(m+1))^(1/2)))+((L*(m+1))^
(1/2)))*(((m+1)/2)+((2)/4)*((a1/L)^(1/2))))-...

b1*(((m+1)+((b0*((2)^2))/2))*erfc(((2)/2)*((b0/(m+1))^(1/2)))-
(2)*((b0)^(1/2))*(((m+1)/pi)^(1/2))*exp(-(b0*((2)^2))/(4*(m+1)))));

  end
end
  zz=zu;
end

%-----------------------------------------------------------------
```
